# Delayed gastric emptying following pancreatoduodenectomy with alimentary reconstruction according to Roux-en-Y or Billroth-II

**DOI:** 10.1186/s12893-017-0226-x

**Published:** 2017-03-20

**Authors:** Tim R. Glowka, Markus Webler, Hanno Matthaei, Nico Schäfer, Volker Schmitz, Jörg C. Kalff, Jens Standop, Steffen Manekeller

**Affiliations:** 1 Department of Surgery, University of Bonn, Sigmund-Freud-Str. 25, 53105 Bonn, Germany; 2 Department of Orthopedic and Trauma Surgery, University of Bonn, Sigmund-Freud-Str. 25, 53105 Bonn, Germany; 3 Department of Gastroenterology, St. Marienwörth Hospital, Mühlenstr. 39, 55543 Bad Kreuznach, Germany; 4 Department of Surgery, Maria Stern Hospital, Am Anger 1, 53424 Remagen, Germany

**Keywords:** Delayed gastric emptying, DGE, Pancreatoduodenectomy, Billroth II, Whipple, Roux-en-Y

## Abstract

**Background:**

Delayed gastric emptying (DGE) remains the most frequent complication following pancreatoduodenectomy (PD) with published incidences as high as 61%. The present study investigates the impact of bowel reconstruction techniques on DGE following classic PD (Whipple-Kausch procedure) with pancreatogastrostomy (PG).

**Methods:**

We included 168 consecutive patients who underwent PD with PG with either Billroth II type (BII, *n* = 78) or Roux-en-Y type reconstruction (ReY, *n* = 90) between 2004 and 2015. Excluded were patients with conventional single loop reconstruction after pylorus preserving procedures. DGE was classified according to the 2007 International Study Group of Pancreatic Surgery definition. Patients were analyzed regarding severity of DGE, morbidity and mortality, length of hospital stay and demographic factors.

**Results:**

No difference was observed between BII and ReY regarding frequency of DGE. Overall rate for clinically relevant DGE was 30% (ReY) and 26% (BII). BII and ReY did not differ in terms of demographics, morbidity or mortality. DGE significantly prolongs ICU (four vs. two days) and hospital stay (20.5 vs. 14.5 days). Risk factors for DGE development are advanced age, retrocolic reconstruction, postoperative hemorrhage and major complications.

**Conclusions:**

The occurrence of DGE can not be influenced by the type of alimentary reconstruction (ReY vs. BII) following classic PD with PG. Old age and major complications could be identified as important risk factors in multivariate analysis.

**Trial registration:**

German Clinical Trials Register (DRKS) DRKS00011860. Registered 14 March 2017.

## Background

Pancreatoduodenectomy (PD) is the standard surgical procedure for malignant pancreatic head and periampullary tumors [1]. In specialized centers, the surgery can be performed with a relatively low mortality rate of 0–6% [2–4]. Nevertheless, the morbidity rate remains high, ranging from 30% to above 50% [5]. Apart from pancreatic fistula as the most frequent *major* complication following PD [6], delayed gastric emptying (DGE) is even more common with up to 61% reported rates [5, 7]. The type of reconstruction technique after PD is considered to influence the frequency of DGE. While antecolic position of the gastro-/duodenojejunal loop has been considered superior in terms of DGE [8, 9], recent studies demonstrated comparable benefits of retrocolic reconstruction [7, 10, 11]. In terms of DGE frequency, this could also be shown for pylorus-preserving PD compared to classic PD with antrectomy (Kausch-Whipple procedure) [12]. However, in recent years, pylorus resection without antrectomy has been increasingly advocated [13–15]. Furthermore, regarding DGE, single loop (“conventional reconstruction”) and Roux-en-Y (dual loop) reconstruction show no difference [16].

Classic PD with pyloric resection and reconstruction according to Billroth II (BII) and Roux-en-Y (ReY) as standard procedures are performed with decreasing frequency since single loop reconstruction methods and pyloric preservation have proven comparable in terms of fistula formation and DGE with reduced surgery duration and blood loss [8, 17]. However, antral resection with BII or ReY reconstructions are still performed in case of local tumor infiltration to the distal stomach. Apart from the above mentionend perioperative options, Whipple-Kausch procedure as well as pylorus-preserving single-loop PD are equally effective in the treatment of periampullary malignancies [18]. Outside tertiary referral centers, BII and ReY remain in use, but only limited data are available on the incidence of DGE when comparing BII and ReY following PD. To our knowledge, only one study compared BII and ReY reconstructions after pancreatojejunal anastomosis for their impact on DGE [19]. To date, these two reconstruction methods have not been compared after pancreatogastrostomy (PG).

## Methods

**Fig. 1:**
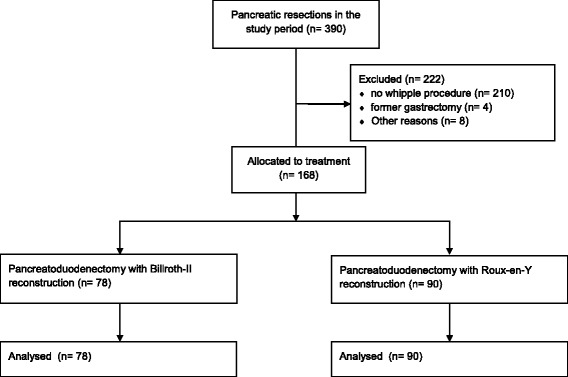
Patients flow chart

Between 2004 and 2015, 390 patients underwent anatomical pancreatic resection at our department. Of these, 168 patients underwent a classic pancreatoduodenectomy with antral resection and reconstruction according to BII or ReY. Excluded were patients with pylorus preserving procedures and conventional reconstruction with a single jejunal loop, and patients who had previous gastrectomy (Fig. 1). All pancreatic resections were prospectively recorded in a pancreatic resection database with the approval of the institutional ethics committee (Ethik-Kommission der Medizinischen Fakultät der Rheinischen Friedrich-Wilhelms-Universität, 347/13) and with obtaining written informed consent from the participants. Morbidity and mortality were consistently documented according to the Dindo-Clavien- classification [20].

Perioperative management was conducted according to an institutional recovery programm: sip feeds were provided in case of preoperative malnutrition; parenteral nutrition was only administered when the oral route was inaccesible. No endoscopic biliary drainage was performed if serum bilirubin was below 250 μmol/l and surgery was scheduled within the next ten days. No oral bowel preparation was used and oral fasting was limited to 2 h for liquids and 6 h for solids. A mid-thoracic epidural catheter was placed by default, while in case of contraindications, missing placement options or catheter disfunction, patient-controlled analgesia was considered as alternative. Anesthesia was carried out according to guidelines (postoperative nausea and vomiting prophylaxis if required, near zero fluid balance, tranfusion according to patient blood mangement guidelines and close glycemic control).

**Fig. 2:**
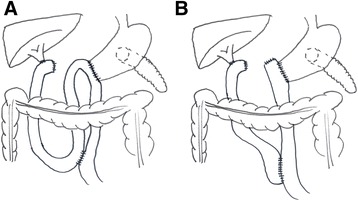
Schematic drawing of pancreatoduodenectomy with Billroth-II reconstruction (**a**) and Roux-en-Y reconstruction (**b**)

PD was performed via a bilateral subcostal incision. After complete abdominal exploration and exclusion of arterial infiltation, PD was carried out with antrectomy, standard lymphadenectomy by default and PG as previously described [21]. Infiltration to the portal or superior mesenteric vene was resected en-bloc with the pancreas. If simple suture led to narrowing of the vein, resection and end-to-end anastomosis was performed. Choledochojejunostomy was carried out to the oral jejunal loop with a retrocolic single-layer end-to-side running suture (4/0 absorbable). Reconstruction method was chosen in a pragmatic manner according to the surgeon’s preference [22]. In BII reconstruction, a double layer end-to-side running suture gastrojejunostomy (4/0 absorbable) was performed 40 cm aboral to the biliary anstomosis, while 15 cm below, reconstruction was completed by a (stapled) Braun enteroenterostomy (Fig. 2a). ReY reconstruction was performed with the same gastrojejunal anstomosis with an isolated jejunal loop and enteroenterostomy 30 cm aborally (Fig. 2b). Two soft drains were placed at the sites of PG and choledochojejunostomy before closure of the abdomen. These drains were removed between postoperative days (POD) 3–5 if no elevated amylase content (compared to serum amylase) could be detected in measurements. By default, all patients spent at least one night in the intensive care unit. A 14 French nasogastric tube (NGT) was placed and subsequently removed on POD 3 when output fell below 500 ml/day. Patients were allowed to drink water on the day of surgery, liquid diet was introduced from POD 2, and solid food from POD 3 and increased according to a standard protocol (POD 3 fat reduced/easily digestible, POD 4 fiber reduced/easily digestible, POD 5 basic diet (no pulses/no brassica), POD 6 normal diet). If no bowel movement had occurred by POD 3, oral laxative (magnesium sulfate) was administered. Transition to a normal diet was discontinued in case of vomiting. All patients received perioperative antibiotic prophylaxis (aminopenicillin plus β-lactamase inhibitor) and weight-adapted thrombosis prophylaxis (continued for four weeks after surgery plus support stockings) but no secretion inhibitor (octreotide) on a regular basis. DGE was recorded as stipulated by the 2007 International Study Group of Pancreatic Surgery (ISGPS) definition [5]. Based on duration of NGT, need for reinsertion, the day, when solid food was first tolerated, occurrence of vomiting and use of prokinetics, DGE was classified according to three grades. Since the ISGPS definition tends to overestimate DGE at °A [23], some authors report the clinically relevant °B and °C when specific treatment is indicated. Prior to 2007, patients were retrospectively graded according to the ISGPS definition based on their medical records.

Data were recorded and analyzed with Excel 2013 (Microsoft Corporation, Redmond, Washington, USA) and SPSS 23 (IBM Corporation, Armonk, New York, USA). Continuously and normally distributed variables were expressed as medians ± standard deviation and analyzed using Student’s *t* test, while non-normally distributed data was expressed as medians and interquartile range and analyzed using the Mann–Whitney *U* test. Categorical data was expressed as proportions and compared with the Pearson *χ* ^2^ or the Fisher’s exact test as appropriate. Factors with *P* <0.1 in the univariate analysis were included in multivariate stepwise logistic regression analysis. The relative risk was described by the estimated odds ratio with 95% confidence intervals. A *P*-value <0.05 was considered statistically significant.

## Results

ReY and BII groups were comparable in age, gender, diagnosis and preoperative characteristics. Intra- and perioperative data were equal. There was no difference between morbidity factors or mortality. Clinically significant DGE occurred in 30% (*n* = 27, ReY) and 26% (*n* = 20, BII), respectively (Table 1). Patients suffering from DGE were significantly older (68 vs. 62 years), while no significant difference in other demographic factors, such as diagnosis or preoperative risk factors, could be shown (Table 2). Surgery duration and blood loss did not differ in patients with and without DGE (Table 3). In the DGE group, more patients were reconstructed with a retrocolic gastrojejunostomy (89/98 (91%) vs. 66/80 (80%), *P* = 0.047) and ICU stay (four vs. two days, *P* < 0.001) as well as hospital stay (20.5 vs. 14.5 days, *P* < 0.001) were significantly longer. Major complications (Dindo-Clavien °3-5) were associated with DGE (42% vs. 23%, *P* = 0.01), while pancreatic fistula was only slightly more common in the DGE group compared with patients not suffering from DGE (30% vs. 38%, *P* = 0.297). Secondary DGE (following other intraabdominal complications) was more common than primary DGE (56 vs. 42, *P* = 0.068) and DGE was more severe in secondary DGE (°A 25 vs. 26, *P* = 0.971; °B 9 vs. 11, *P* = 0.717; °C 8 vs. 19, *P* = 0.030). Significantly more patients with DGE suffered from post pancreatectomy hemorrhages (PPH; 28% vs. 14%, *P* = 0.041), which was also a risk factor for the severity of DGE (°C 12/37 vs. 15/131 (PPH yes/no), *P* = 0.002). If no DGE developed, solid food was tolerated on average on POD 6 and NGT was then removed on POD 2 (Table 4). If DGE developed, solid food was tolerated on POD 11 (*P* < 0.001), NGT removal occurred on POD 4 (*P* < 0.001) and NGT reinsertion was required in 39% of the patients (*P* < 0.001). Vomiting and use of prokinetics were significantly more common in the DGE group. DGE was graded as °A in 52%, °B in 20% and °C in 28% of the patients. In univariate analysis, the following factors qualified for multivariate analysis: patient age (dichotomized for multivariate analysis), weight loss, cholangitis, antecolic reconstruction, extended lymphadenectomy, PPH and major complications (Table 5). Age above 70 years (*P* = 0.009) and major complications (*P* = 0.003) proved to be significant risk factors in multivariate analysis.

**Table 1:** Preoperative and perioperative characteristics

		ReY		BII		*P*
		*n* = 90		*n* = 78		
Age, years		65 (55–74)		67 (54–70)		0.948
Gender						0.092
female		29	(32%)	35	(45%)	
male		61	(68%)	43	(55%)	
BMI		25,2 ± 3,5		23,4 ± 4,3		0.066
Diagnosis						
Malignant		71	(79%)	59	(76%)	0.616
Ductal adenocarcinoma		39	(43%)	32	(41%)	
Ampullary carcinoma		13	(14%)	16	(21%)	
Distal bile duct carcinoma		8	(9%)	8	(10%)	
Benign		19	(21%)	19	(24%)	
Pancreatitis		11	(12%)	17	(22%)	
DM	pre	14	(16%)	17	(22%)	0.298
	post	14	(16%)	21	(27%)	0.076
Alcohol		23	(26%)	15	(19%)	0.328
Smoker		43	(48%)	26	(33%)	0.058
Weight loss		39	(43%)	25	(32%)	0.133
Preoperative biliary drainage		56	(62%)	45	(58%)	0.618
Cholangitis		21	(23%)	16	(21%)	0.66
Time of operation	min	434 ± 104		410 ± 77		0.104
Red blood cell transfusion	units	2 (0–4)		2 (1,5-4)		0.518
Blood loss		1000 (500–1600)		800 (400–1300)		0.262
Clavien classification						0.145
minor		55	(61%)	56	(72%)	
major		35	(39%)	22	(28%)	
Mortality		1	(1%)	1	(1%)	1.0
Pancreatic fistula		36	(40%)	22	(28%)	0.109
Post pancretectomy hemorrhage		20	(22%)	17	(22%)	0.947
DGE		49	(54%)	49	(63%)	0.272
	B/C	27	(30%)	20	(26%)	0.53

Data are expressed as mean ± SD, *number (%), or* median (interquartile range)

**Table 2:** Preoperative Characteristics

		No DGE		DGE		*P*
		*n* = 70		*n* = 98		
Age, years		62 (51–69)		68 (60–74)		*0.003*
Gender						0.39
female		24	(34%)	40	(41%)	
male		46	(66%)	58	(59%)	
BMI		24,6 ± 3,8		24,4 ± 4		0.888
Diagnosis						
Malignant		54	(77%)	76	(78%)	0.95
Ductal adenocarcinoma		31	(44%)	40	(41%)	
Ampullary carcinoma		10	(14%)	19	(19%)	
Distal bile duct carcinoma		7	(10%)	9	(9%)	
Benign		16	(23%)	22	(22%)	
Pancreatitis		13	(19%)	15	(15%)	
DM	pre	12	(17%)	19	(19%)	0.712
	post	15	(21%)	20	(20%)	0.835
Alcohol		19	(27%)	19	(19%)	0.236
Smoker		31	(44%)	38	(39%)	0.474
Weight loss		32	(46%)	32	(33%)	0.086
Preoperative biliary drainage		43	(61%)	58	(59%)	0.831
Cholangitis		20	(29%)	17	(17%)	0.083

Data are expressed as mean ± SD, *number (%), or* median (interquartile range). Statistical significance indicated by italics

**Table 3:** Perioperative characteristics and morbidity

		No DGE		DGE		*P*
		*n* = 70		*n* = 98		
Time of operation	min	427 ± 90		421 ± 97		0.721
Red blood cell transfusion	units	2 (0–4)		2 (0–4)		0.091
Blood loss	ml	750 (500–1500)		1000 (400–1800)		0.324
Antecolic reconstruction		14	(20%)	9	(9%)	*0.047*
Extended lymphadenectomy		31	(44%)	31	(32%)	0.094
Venous resection		4	(6%)	13	(13%)	0.11
Roux-en-Y reconstruction		41	(59%)	49	(50%)	0.272
ICU stay	days	2 (2–4)		4 (3–7)		*<0.001*
Primary DGE				42		0.068
Secondary DGE				56		
Clavien classification						
minor		54	(77%)	57	(58%)	
major		16	(23%)	41	(42%)	*0.010*
Mortality		0	(0%)	2	(2%)	0.511
Redo operation		10	(14%)	24	(24%)	0.105
Pancreatic fistula		21	(30%)	37	(38%)	0.297
A		13	(19%)	24	(25%)	0.361
B		3	(4%)	5	(5%)	1.0
C		5	(7%)	8	(8%)	0.807
Post pancreatectomy hemorrhage		10	(14%)	27	(28%)	*0.041*
Wound infection		9	(13%)	17	(14%)	0.428
Intraabdominal abscess formation		6	(9%)	9	(9%)	0.891
Hospital stay	days	14,5 (13–21,5)		20,5 (16–30)		*<0,001*

Data are expressed as mean ± SD, *number (%), or median (interqu*artile range). Statistical significance indicated by italics

**Table 4:** DGE and DGE-related parameters

	No DGE		DGE		*P*
	*n* = 70		*n* = 98		
Tolerate solid diet (days)	6 (5–6,25)		11 (8–15)		*<0.001*
Nasogastric tube (NGT)					
NGT duration (days)	2 (1–3)		4 (2,75-5,25)		*<0.001*
NGT reinsertion	5	(7%)	38	(39%)	*<0.001*
Vomiting	14	(20%)	49	(50%)	*<0.001*
Use of prokinetics	16	(23%)	61	(62%)	*<0.001*
DGE °A			51	(52%)	
DGE °B			20	(20%)	
DGE °C			27	(28%)	

Data are expressed as mean ± SD, number (%), or median (interquartile range). Statistical significance indicated by italic

**Table 5:** Risk factors for DGE

	Odds ratio	95% CI	*P*
univariate			
Age >70 years	2.323	1.136 – 4.749	*0.019*
Weight loss	0.576	0.306 – 1.083	0.086
Cholangitis	0.525	0.251 – 1.096	0.083
Antecolic reconstruction	0.409	0.166 – 1.008	*0.047*
Extended lymphadenectomy	0.582	0.308 – 1.099	0.094
Post pancreatectomy hemorrhage	2.282	1.022 – 5.092	*0.041*
Major complications (Dindo-Clavien °3-5)	2.428	1.221 – 4.827	*0.01*
multivariate			
Age >70 years	2.745	1.29 – 5.841	*0.009*
Major complications (Dindo-Clavien °3-5)	3.03	1.458 – 6.297	*0.003*

*CI* confidence interval. Statistical significance indicated by italic

## Discussion

Delayed gastic emptying is the most common complication following pancreatoduodenectomy (PD), occuring in 19–61% of patients [5, 7]. Since the first description of DGE following PD by Warshaw in 1985 [24], many attempts have been made to further understand the mechanisms leading to DGE. Proposed factors are a decrease of plasma motlin levels due to resection of the duodenum, ischemia and denervation of the stomach due to mobilisation and lymphadenectomy, or DGE caused by postoperative intra-abdominal complications [25]. Only limited data exist on the effect of dual loop reconstruction on DGE formation, with DGE occurrence ranging from 9.5 to 72% [26–29]. At our department, as in most centers for pancreatic surgery, pylorus-preserving PD with single loop reconstruction is the established standard procedure due to reduced surgery duration and blood loss and equal complication rates [8, 17]. Nevertheless, in case of tumor infiltration to the distal stomach, or after previous gastrectomy, classic PD with dual loop reconstruction is required. Very little is known about the effect of BII and ReY reconstruction on DGE. In 2015, a meta-analysis comparing ReY and BII reconstruction after PD found that DGE frequency can be lowered when using BII reconstruction [30]. A limitation of this study was the different understanding of the surgical reconstruction methods. Two studies compared conventional single loop reconstruction with ReY reconstruction [29, 31], while only one study intentionally compared ReY and BII, again favoring BII reconstruction [19]. However, differences regarding the local setting (e.g. overall length of hospital stay) make their and our findings difficult to compare. Moreover, the authors based their findings on pancreatojejunostomy (PJ) as pancreato-enteric anastomosis. The existing studies did not find a difference in DGE frequency between PJ and PG [4, 32]. However, in these studies, reconstruction was neither specified or performed as conventional single loop reconstruction. Thus, especially after PG, knowledge about DGE after dual loop reconstruction is very limited. In our study, we identified PPH rather than pancreatic fistula as a significant factor contributing to DGE. Most studies comparing PG and PJ found no difference in PPH frequency [33–35], whereas the biggest randomized study, involving 440 patients, found PPH more common after PG [4]. In fact, it was found to be more than twice as common (PJ 11% vs. PG 21%), which is exactly the PPH frequency we observed. Most bleedings (PPH °A 3/0, °B 18/8 and °C 6/2 [DGE yes/no]) were °A/B, which in most cases, could be treated conservatively or endoscopically. The option of easy endoscopic access is one of the advantages of PG reconstruction compared to PJ, making intraluminal PPH easily treatable with interventional gastroscopy [36]. Endoscopic access in PPH after PD with dual loop reconstruction using PJ is more difficult. Other advantages claimed for PG over PJ after PD is a reduced rate of pancreatic and bile leakage [32]. However, the afore mentioned German multicenter trial (RECOPANC) could not confirm this finding [4]. Apart from the treatment of acute postoperative bleeding, long term endoscopic access is still under debate: successful endoscopic retrograde cholangiography is more likely to be achieved after BII than after ReY reconstruction [37]. However, following distal gastrectomy, ReY was found superior to BII in terms of related symptoms, weight gain, as well as regarding endoscopic findings and bile reflux [38]. For PD, no long-term endoscopic examinations exist. Therefore, BII and ReY reconstruction have certain advantages and disadvantages. Both procedures have the same DGE frequency following PD. In our department, BII reconstruction with a Braun enterostomy is performed by default. A recent assessment of Braun enterostomy after PD found it to be beneficial in lowering DGE frequency [39, 40]. In our opinion, Braun enterostomy is obligatory after antrectomy (or subtotal gastrectomy) to prevent biliary reflux, ulceration and long-term impairments associated with subtotal gastrectomy (especially gastric stump carcinoma). In our cohort, patient age was identified as a uni- and multivariate risk factor for DGE. The impact of age on morbidity and mortality after PD varies [41]. Two nationwide surveys from the US and the Netherlands found more complications and a higher morbidity in the elderly [42, 43]. When DGE occurs, ICU stay as well as general hospital stay as markers for health care costs are signifcantly prolonged, while complications after pancreatic surgery generally lead to a cost increase [44]. In today’s age of diagnosis-related groups with case-related reimbursement, prophylaxis of DGE is also of important economic interest. In Germany, PD can only be performed cost-neutrally when the complication rate is low [45]. Therefore, prevention of DGE after PD is not only of major medical, but also economical interest. As DGE is more severe following other intraabdominal complications, thus requiring a longer hospital stay, intraabdominal complications should be avoided as a matter of priority. In particular, secure hemostasis at the pancreatic surface, safe closure of resected vessels (gastroduodenal artery) by non-resorbable sutures and standardized pancreatic anastomosis technique are the cornerstones following pancreatic resections [46]. In the therapy of DGE, it is important to distinguish DGE from postoperative ileus and to rule out mechanical obstruction as previosly described [47]. When DGE is diagnosed, first therapy steps include NGT and prokinetics (erythromycin) [25]. When secondary DGE occurs, the treatment of the underlying cause must be top prioritiy. If DGE persists after the complication was properly treated or in case of longer lasting primary DGE, we recommend endoscopic insertion of a jejunal feeding tube, followed by low-dose (20 mL/h) enteral feeding. In our experience, DGE will then resolve within a few days. This is especially beneficial if nutritional support commences within ten postoperative days [48]. Routine placement of a jejunal tube during surgery can not be recommended at present [49].

## Conclusions

When antrectomy and subsequent dual loop reconstruction is necessary, DGE frequency is equal to pylorus-preserving procedures. DGE occurrence can not be influenced by either BII or ReY reconstruction. Since patient age can not be modified, the primary focus should be to lower postoperative complications. In particular, PPH should be prevented through extensive hemostasis at the pancreatic remnant and the sourrounding vessels. Anteoclic gastrojejunostomy, if technically possible, was helpful in our cohort to further reduce DGE.

## Abbreviations

BII: Billroth-II
DGE: Delayed gastric emptying
ISGPS: International Study Group of Pancreatic Surgery
NGT: Nasogastric tube
PD: Pancreatoduodenectomy
PG: Pancreatogastrostomy
PJ: Pancreatojejunostomy
POD: Psotoperative day
PPH: Post pancreatectomy hemorrhage
ReY: Roux-en-Y
